# Enamel matrix derivative in the treatment of tooth replantation: from a biological basis to clinical application

**DOI:** 10.1080/07853890.2024.2424452

**Published:** 2024-11-09

**Authors:** Yao Lin, Liangping Chen, Yuling Xu, Mingwei Xu, Qinghua Liu, Junbing He

**Affiliations:** a Jieyang Medical Research Center, Jieyang People’s Hospital, Jieyang, Guangdong, China; b The Department of Stomatology, Jieyang People’s Hospital, Jieyang, Guangdong, China; c The Medical Supplies Room, Guigang City health Service Center, Guigang, Guangxi, China

**Keywords:** Enamel matrix derivative, replanted teeth, periodontal healing, root adsorption, survival

## Abstract

**Background:**

An efficient therapeutic strategy for patients with replanted teeth has been extremely challenging because complete displacement of the teeth is inevitably accompanied by severe damage to periodontal tissue. Enamel matrix derivative (EMD) shows promise for periodontal regeneration, but its effects on replanted teeth remain unknown. This study systematically summarized the biological basis of EMD in replantation dental therapy and assessed its effect on clinical prognosis.

**Methods:**

Potential studies were searched *via* the Cochrane Library, Web of Science and PubMed databases from inception to November 23, 2023.

**Results:**

A total of 329 patients with 375 replanted teeth met the inclusion criteria. Our pooling results indicated that EMD did not provide a numerical advantage for restoring normal PDL healing in replanted teeth (RR=0.38, *P*=0.161). A significantly lower extraction risk was observed in EMD-treated group than non-EMD-treated group (RR=0.47, *P*=0.001). Moreover, the survival rate of replanted teeth with root resorption was significantly increased by the application of EMD (RR=2.21, *P*=0.017). Although the pooled outcomes suggested increased incidence of surface resorption (RR=1.19, *P*=0.730) and decreased risk of inflammation resorption (RR=0.68, *P*=0.560) among replanted teeth with resorption in the EMD-treated group, neither difference was statistically significant.

**Conclusion:**

For patients with replanted teeth, EMD treatment may not result in a numerical increase in normal PDL healing. However, as a biological regulator, EMD may arrest the progression of resorption, thus reducing the risk of extraction in the early stage. Well-designed randomized trials are required to validate these results due to the poor quality of evidence.

## Introduction

Enamel matrix proteins (EMPs), which are formed by Hertwig’s epithelial sheath and are responsible for initiating root development, are considered crucial stimulating factors in the process of cementogenesis and the periodontal attachment apparatus [1,2]. The commercial enamel product enamel matrix derivative/emdogain (EMD) was derived from developing porcine embryonal enamel and has been the basis of numerous publications investigating its future use in periodontal regeneration [3].

**Figure 1.**
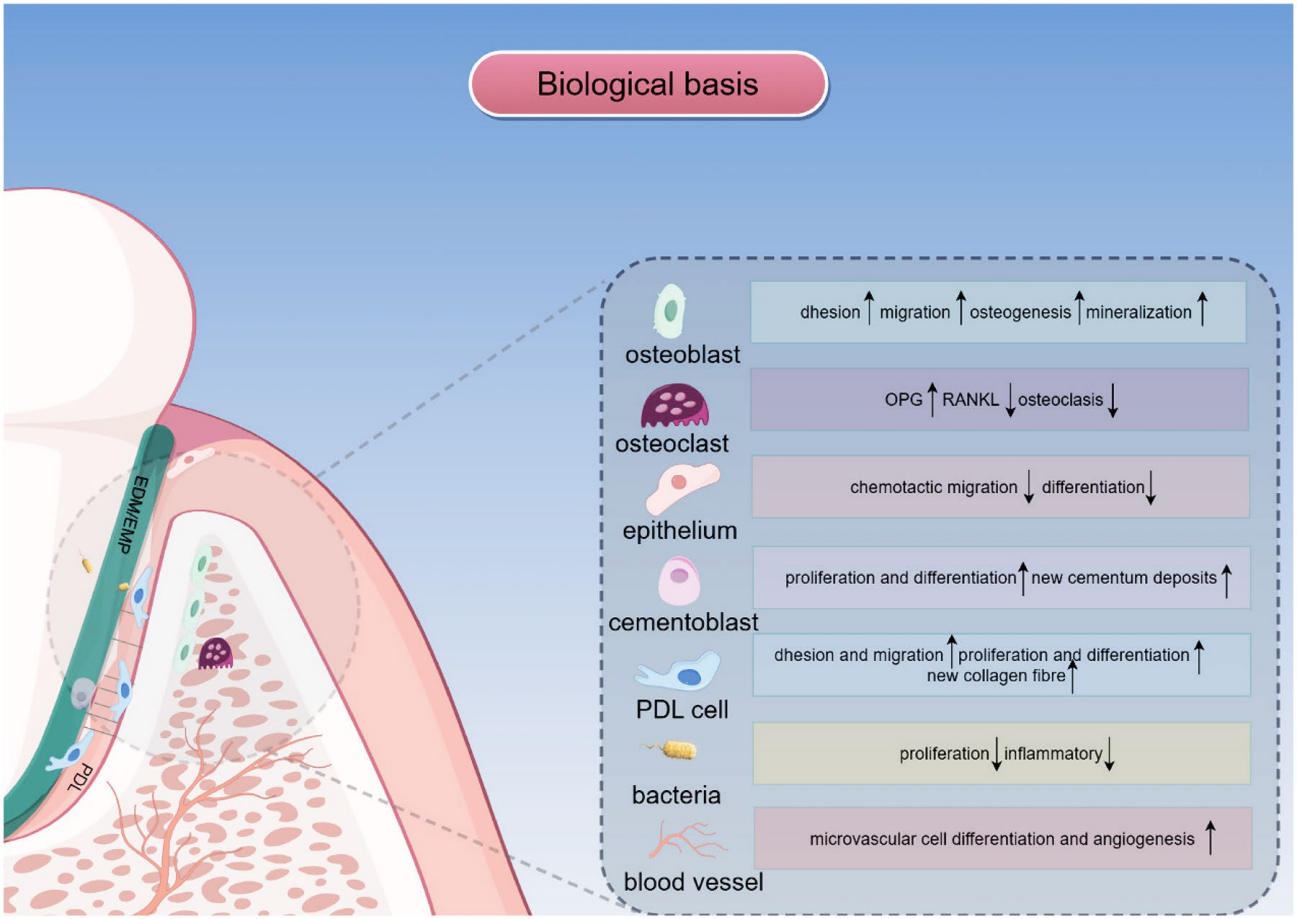
The graph shows that the use of EMD influences the biological behaviour of cells, which is the biological basis for repairing damaged periodontal tissue after replantation. EMD promotes the migration and adhesion of osteoblasts to injured periodontal tissues and facilitates osteogenic differentiation and mineralization. EMD regulated the expression of RANKL balance, leading to the inhibition of resorbing activity of osteoclasts. EMD also prevented the rapid growth of the gingival epithelium, thus providing more opportunities and sites for periodontal membrane regeneration. Furthermore, EMD mediates the formation of acellular cementum on the root of the developing tooth, providing a foundation for all necessary tissues associated with functional periodontal attachment. Moreover, periodontal membrane cells promote the production of more collagen fibres that insert into the neocementum and span the periodontal apparatus. Research has also revealed that EMD has antibacterial and anti-inflammatory effects. In addition, EMD improves endothelial cell proliferation and increases local microvascular density in periodontal tissue. The formation of new blood vessels increases the tissue blood supply, which accelerates the process of healing.

Regenerative therapy often requires the attachment of new connective tissue to the root surface. It is important to underline in this process that it is not only collagen fibres that matter but also the neocementum. Periodontal fibres were embedded during the deposition of new cementum and thereby formed new attachments. Hence, promoting the occupation of the root surface by periodontal ligament (PDL) cells is particularly important. Unfortunately, the gingival epithelium grows the fastest in the periodontal tissue, and even worse, it inhibits the function of periodontal ligament cells once they reach the root surface. It has been demonstrated that amelogenin (AM), a type of adhesion molecule that constitutes the main component of enamel matrix proteins, can promote the adhesion and extension of cells in the early stage. Notably, this biological effect is cell specific, as EMPs can significantly promote the adhesion and migration of periodontal ligament cells, gingival fibroblasts, bone marrow stromal cells, osteoblasts, and bone cells [4–6] but exhibit an inhibitory effect on gingival epithelial cells [7]. Thus, the application of EMPs may provide more opportunities to reconstruct new attachments. During the periodontal regeneration process, the inflammatory environment and hyperactivity of osteoclasts may contribute to the suppression of periodontal cell differentiation and bone resorption, thereby limiting regeneration of the periodontium. EMP was proven to have bacteriostatic and anti-inflammatory properties. At the molecular level, early researchers suggested that EMP downregulated the expression of genes involved in inflammatory events early in wound healing, while genes encoding molecules that promote growth and repair were upregulated [8]. Similarly, a recent study revealed the inhibition of TNF-α release and the downregulation of inflammatory factor expression in human epithelial cells and osteoblasts stimulated with bacterial lipopolysaccharides and EMD [9]. In addition, enamel protein was shown to suppress root resorption, RANKL production and osteoclast formation [10,11]. Moreover, at appropriate concentrations, EMD can promote osteogenic, mineralization, and angiogenic differentiation under environmental conditions *in vitro* [12,13]. Based on the above biological background, where cells are exposed to EMD and its potential beneficial effect on periodontal wound healing and regeneration has emerged, EMD is considered a superior material with clinical application prospects (Figure 1).

Accidental tooth avulsion is a traumatic dental injury that causes the entire loss of teeth with long-term treatment burden. Tooth replantation is typically carried out in cases of traumatic tooth avulsion, as well as intentional tooth extraction for *ex vivo* dental therapy (e.g. endodontic treatment); however, the prognosis often remains as unpredictable. Periodontal healing appears to be most likely unsuccessful when the periodontal ligament (PDL) is severely damaged. Despite the ‘advances’ in treatment for tooth avulsion in the last decade, replantation outcomes have not improved. Müller et al. reported only 65% of replanted avulsed teeth survived in an average observation duration of 3.5 years [14]. Other data indicated survival rates ranged from 50% to 83% of replanted avulsed teeth, underscoring the great challenge of tooth replantation [15–17]. Obviously, alternative an external stimulus for activating the healing process and preventing or delaying resorption under certain condition was necessity. EMD has the biological background of mobilizing the potential of periodontal regeneration and repair, which provides a new idea and opportunity to break the limit of the *in vitro* survival time of the dislocated teeth.

Here, a relevant question arises: Can it play a powerful biological role in promoting healing under traumatic conditions? Some early animal experimental findings demonstrated that EMD was a bioactive root conditioning agent for reintegration of avulsed teeth [18], but subsequent research showed that EMD was unable to stimulate tissue repair in replanted teeth [19,20]. However, Sahng G. Kim et al. performed a meta-analysis with six animal studies and revealed that EMD led to greater normal periodontal healing and surface root resorption but less replacement root and inflammatory root resorption in the presence of a PDL [21]. This is an initial positive signal as root resorption was still a significant challenge in replantation in clinical, especially progressive replacement and inflammatory resorption which may leads to the early tooth loss. It is worth exploring whether the same effect can be achieved in human trials. More recently, according to the research of Sugaya T et al. EMD was even effective in regenerating cementum on root surfaces from which the periodontal ligament had been lost around the fracture line and in reducing the incidence of root resorption when replanting teeth after a vertical root fracture [22]. Given that animal experiments are encouraged for the treatment of replanted teeth, the clinical efficacy of EMD used by physicians is of interest. Indeed, over the past two decades, clinical trials have been performed to provide further evidence for regeneration in replantation, thus corroborating the findings from animals. In 2001, Filippi A et al. reported that EMD may prevent or delay ankylosis in children with replanted ankylosed teeth [23]. Subsequently, case reports involving two elderly patients also demonstrated that the mean probing attachment height improved by 3.2 and 1.5 mm after tooth transplantation [24]. Periodontal ligament-like space around the root or new alveolar bone surrounding the transplantation tooth was present in periapical radiographs in some of the patients [23–25]. In contrast, another recent study reported that this approach was clinically ineffective [26]. Interestingly, a more recent case series suggested that EMD significantly reduced the probing depth (6.4 ± 2.6 mm), increased the clinical attachment level (5.9 ± 2.5 mm), and increased the radiographic bone level (48.2 ± 26.1%) compared to baseline values. Moreover, clinical healing was observed in 94.12% of the patients, and radiographs revealed no root resorption or ankylosis [27]. However, it should be noted that most of the evidence that recommended the use of EMD in patients with replanted teeth was a single-arm proportion [22–25, 27], and the effect of EMD on patient-centred outcomes remains to be evaluated with increasing evidence.

To date, based on the amount of data available on the functions of EMD for root development, bone formation, and soft tissue repair, the translation of these findings into a clinical application with the aim of improving healing after replantation has attracted increasing interest. Therefore, to assess the clinically beneficial impact of EMD treatment in replanted teeth, we conducted the meta-analysis using all of the evidence that was accessible in the published studies.

## Materials and methods

### Protocol and registration

This study followed the guidelines of Preferred Reporting Items for Systematic Reviews and Meta-Analyses (PRISMA) statement and Cochrane Methodology (**Additional file 1 in Supplementary**) [28,29]. The protocol was registered with INPLASY (202450016) and is available in full at 10.37766/inplasy2024.5.0016.

### PICO question

The PICO framework, which is based on the participants (P), interventions (I), controls (C), outcomes (O), was used to formulate the research question in our meta-analysis as follows:

- Does EMD treatment provide superior clinical outcomes (i.e. periodontal healing, the risk of tooth extraction, survival rate in resorption cases) compared with conventional therapy without EMD in patients with replanted teeth?
- Does EMD affect the type of resorption among replanted teeth with root resorption?

### Inclusion and exclusion criteria

The criteria for inclusion were as follows: I) Patients who have tooth replantation; II) The application of EMD to the root surface of the transplanted teeth in the experimental group; and III) clinical prognosis including survival rate or extraction, periodontal healing or root resorption, to evaluate the effectiveness of EMD; IV) Articles reporting experimental or observational clinical studies investigating the role of EMD in the treatment of tooth replantation. The exclusion criteria were as follows: I) animal or *in vitro* studies; II) duplicated data; and III) clinical trials without controls.

### Search Methodology

Yao Lin and Liangping Chen conducted separate searches in the Cochrane Library, Web of Science, and PubMed databases to identify papers that met the specified inclusion criteria between the launch of the databases and November 23, 2023. The studies included were identified based on text words or Medical Subject Headings (MeSH) in the following manner: replantation [MeSH Terms], avulsion, enamel matrix protein, emdogain, enamel matrix derivative, transplantation, autotransplantation, and replanted teeth. **Additional file 2 in Supplementary** displays the search methodology and query specifics. Additionally, we identified additional relevant publications *via* using Google Scholar and analyzing the reference lists of the obtained papers.

### Study selection criteria and data extraction

Two reviewers (Yao Lin and Liangping Chen) independently screened the titles and abstracts of the articles retrieved from the literature search. Based on the inclusion criteria and the exclusion criteria, the possibly eligible full-text articles were then included for further evaluation. Two independent reviewers extracted the data, including the study ID, first author and year of publication, age, sample size, detail of EMD used, endodontic treatment, clinical outcome, and the authors’ conclusions. Any disagreements between two reviewers were settled through deliberation or evaluation by a third author (Mingwei Xu).

### Methodological quality and level of evidence

The quality and risk of bias assessment were carried out based on the Newcastle Ottawa Scale (NOS) for nonrandomized studies. The scale employs a star system to evaluate three broad categories, including the realms of selection, comparisons, and results [30]. Each category in the NOS scale comprised multiple entries, together accumulating a score of 9 points. The NOS scores were assigned thresholds of 7-9, 4-6, and 0-3 points, corresponding to high-, moderate-, and low-quality research criteria, respectively. An independent evaluation of the quality was carried out *via* Junbing He and Yuling Xu. Any differences of opinion were settled by agreement with a third author (Qinghua Liu).

### Statistical analysis

To extract and evaluate the data, STATA statistical software (version 15.1) was used. For dichotomous outcomes, the estimated effects were shown as RRs with 95% CIs. Between-study heterogeneity was estimated using the Cochran’s Q statistic and the I^2^ statistic; values of 25%, 50%, and 75%, respectively, are thought to signify low, moderate, and high heterogeneity [31]. The results of the studies with explicit evidence of statistical heterogeneity (significance for I^2^ >50%) were pooled using a random-effects model. For research with lesser statistical heterogeneity, a fixed-effect model was employed to pool the outcomes. Due to the limited number of the included studies, publication bias and sensitivity analysis were conducted for more than 8 studies. A significance level of *p* < 0.05 was considered to demonstrate sufficient statistical significance.

### Evidence confidence

The strength of recommendation evaluations and confidence of evidence (conducted by Junbing He and Yao Lin) were determined according to the guidelines of the Grading of Recommendations Assessment, Development, and Evaluation (GRADE) methodology, which rated the evidence at four levels: low, very low, moderate, and high certainty for each outcome [32].

## Results

### Study selection

**Figure 2.**
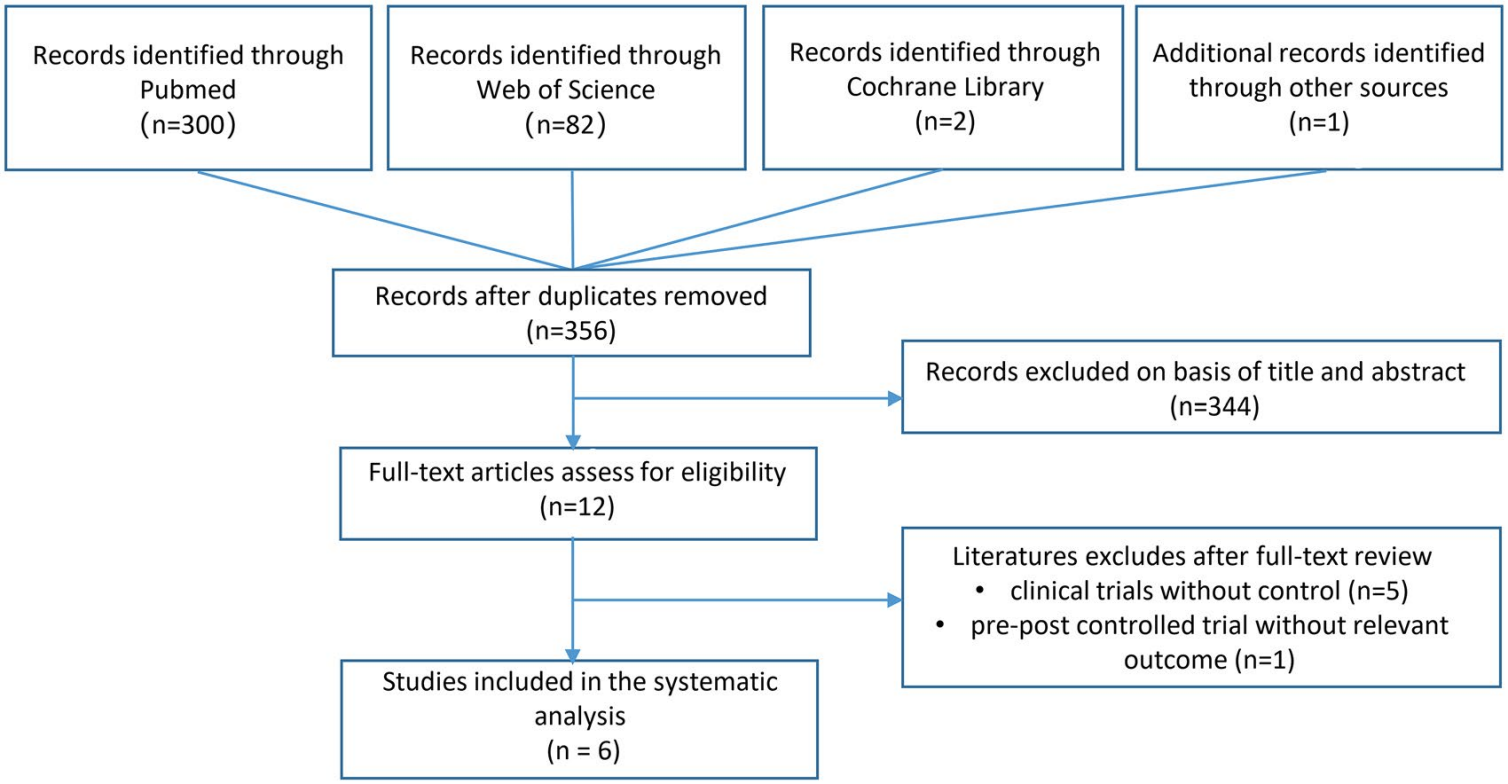
Study inclusion flow chart.

The study inclusion flow chart is shown in Figure 2. The initial literature search yielded a total of 385 records. A total of 300 studies from the PubMed database, 82 from Web of Science, 2 from the Cochrane Library, and 1 from additional research were found. The literature search identified 356 references articles after the removal of duplicates. After screening the title and abstract in accordance with the criteria of inclusion and exclusion, 344 papers were eliminated, leaving 12 articles that were subjected to full-text examination. However, 5 of these remaining studies were excluded because no control groups were included, and 1 study was excluded because of no relevant outcomes. Finally, 6 published studies were included in this systematic review and meta-analysis [33–38].

### Study characteristics

The characteristics of the included studies are summarized in Table 1. All of the selected articles were nonrandomized clinical trials [33–38]. The included articles were all published from 2000 onwards and varied from 2005-2021, three studies were published in 2005. The mean age was not provided in one study [37], and it ranged between 12 and 44.3 years in the remaining published studies [33–36, 38]. All studies were performed in patients with permanent replanted teeth following accidental tooth avulsion [33, 35,36, 38], or intentional tooth extraction [34, 37]. The mean follow-up periods ranged from approximately 3 to 33.6 months in four studies [33, 35, 37,38]. However, the mean follow-up data were not available in two studies [34, 36]; the median follow-up period was 2.8 years in one of the studies [36], and the replanted teeth were followed up for a period of 6-120 months in the remaining study [34]. Most of the included studies [34–36, 38] treated the teeth endodontically, whereas 17.8% [33] or 50.0% of replanted teeth [37] did not receive endodontic therapy. Replanted teeth were placed into a special storage medium (DentoSafe) before surgery in most of the studies [33, 35,36]. The experimental tooth was covered with EMD prior to replantation, and the alveolar socket was filled with EMD except for one study [34] that EMD was only delivered to the root surface. The number of teeth retained in the oral cavity after therapy was reported in five studies [33–37]. The data on periodontal healing were available in five studies [33, 35–38]. However, it should be noted that the EMD treatment group in one study [38] was compared with the control sample from the other studies, in which the sample size was not provided.

**Table 1.** Characteristics of the included studies.

ID	Year	Patient enrollment	Age (years)	Cause of tooth replantation	Storage media	Endodontic treatment	EMD application	Follow-up (month)	Periodontal healing	Resorption n (%) [EX, n]	Risk of extractionn (%)	Authors’ conclusion
Chappuis V [33]	2005	45 avulsed permanent teeth in 34 patients	Mean: 21 Rang: 6-48	Accidental tooth avulsion	Dentosafe	CH	EMD (Straumann) was applied into the socket and onto the root surface	Mean:12	T: 2 (20%); C:24 (68.6%)	Surface: T, 2 (20%); C, 1(2.9%); Replacement: T, 5 (50%); C, 8 (22.8%); Inflammatory: T, 1 (10%); C, 2 (5.47%)	2 (0.4%)	No beneficial effect of using EMD was observed to avoid replacement resorption
Wu SY [34]	2021	215 teeth in 199 patients	Mean: 44.3 Rang: 20-88	Intentional tooth extraction	NR	Super EBA/MTA	EMD (Straumann) was applied to cover the root surface	6-120	NR	NR	T: 37 (19.8%); C: 13 (46.4%)	EMD promote the formation and regeneration of periodontal apparatus, therefore increasing the functioning rate and improving the treatment outcome.
Pohl Y [35]	2005	28 replanted permanent teeth in 24 patients	Mean: 10.3 Rang: 7.1-17.3	Accidental tooth avulsion	Dentosafe/milk/ saline	Extraoral retrograde insertion of post made of ceramics or titanium	EMD (Emdogain) was applied onto the root surface and into the alveolus	Mean: 31.2 Median: 23.8	–	Surface: -; Replacement: T, 7 (50%) [EX, 3]; C, 7 (50%) [EX, 6]; Inflammatory: T, 0 (0%); C, 1 (7.1%) [EX, 1]	T: 3 (21.4%); C: 7 (50%)	Regenerative therapy with EMD might promote healing in teeth with an extraoral non physiologic storage of limited duration.
Werder P [36]	2011	42 replanted permanent incisors in 37 patients	Mean: 16.3 Rang: 6-62	Accidental tooth avulsion	Dentosafe/milk/saline/ other dry	CH/MTA	EMD (Straumann) was additionally applied into the socket and onto the root surface.	Median: 33.6	T: 0 (0%); C: 20 (62.5%);	Surface: T, 0 (0%); C, 1 (3.1%); Replacement: T, 7 (100%) [EX, 1]; C, 11 (34.4%) [EX, 5); Inflammatory: T, 0 (0%); C, 0 (0%)	T: 1 (14.3%); C: 5 (15.6%)	Normal periodontal healing could not be observed in teeth treated with EMD
Fridström M [37]	2008	20 replanted teeth in 10 patients	Mean: NR Rang: 12-15	Intentional tooth extraction	dry	Guttapercha and sealer	The experimental tooth was covered with EMD prior to replantation and the alveolar socket was filled with EMD	Mean: 3	T: 6 (60%); C: 8 (80%)	Surface: T, 2 (20%); C, 1 (10%); Replacement: -; Inflammatory: T, 2 (20%) [EX, 1]; C, 1 (10%) [EX, 1]	T: 1 (10%); C: 1 (10%)	The results were in favor of Emdogain, but the overall difference between the Emdogain-treated tooth and its control was rather small
Barrett EJ [38]	2005	25 avulsed permanent incisors in 25 patients	Mean: 12 Rang: 7.7- 17.6	Accidental tooth avulsion	dry/wet	Guttapercha /sealer	EMD was delivered to the root surface and into the alveolar socket	Mean: 20.6 (T)	T: 10 (40%); C1: NR (5%); C2: NR (10%)	Surface: T, 0 (0%); C1, (5%); C2, (0%); Replacement: T, 15 (60%); C1, NR (55%); C2, NR (65%); Inflammatory: T, 0 (0%); C1, NR (35%); C2, NR (25%)	NR	Although the Emdogain protocol did not produce periodontal regeneration, it did eliminate inflammatory resorption and infection and led to significantly less root resorption compared with the two historical controls.

Note: EMD, enamel matrix derivative; T, test group (EMD); C, control group (No-EMD); CH, calcium hydroxide; MTA, mineral trioxide aggregate; EX, extraction; NR, not report; -: not applicable.

### Quality evaluation

None of the included studies fulfilled all the NOS indicators. The most common methodological limitation was bias due to confounding, as individuals who were not matched in the design and/or confounders were unadjusted for analysis in most of the included studies [33–36, 38]. Although independent or blinded assessments of outcomes were reported in only two papers [34, 38], the outcomes were confirmed by reference to radiographs in all of the studies. The quality evaluation scores varied between 6 and 8 on a scale of maximum 9 (**Additional file 3 in Supplementary**).

### Periodontal healing at the longest follow-up

**Figure 3.**
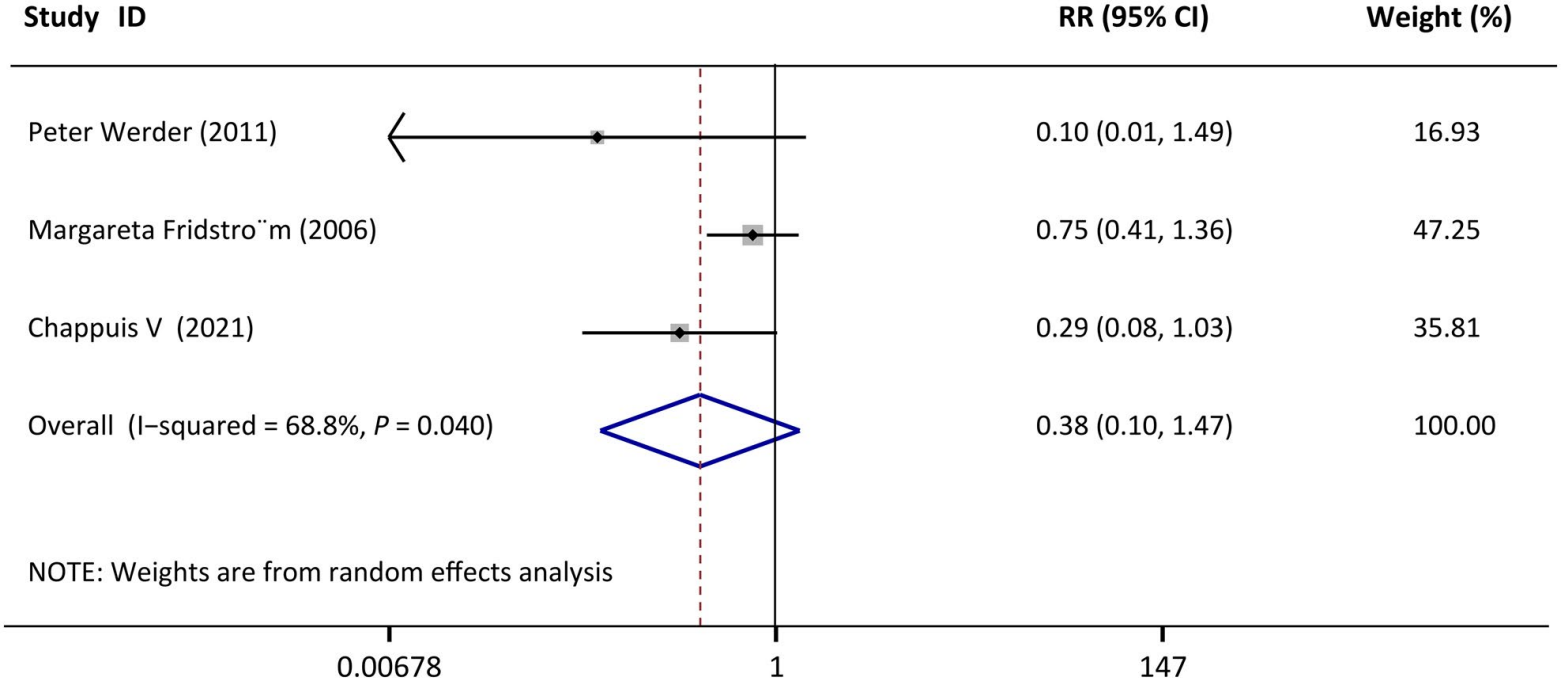
Forest plots of comparisons: EMD versus no-EMD; outcomes: periodontal healing at longest follow-up.

The healing patterns were reported in most of the published studies [33, 35–38]; of these, three studies consistently categorized healing after replantation as normal periodontal healing, surface resorption, inflammatory resorption, and replacement resorption according to clinical and radiographic exams [33, 36,37]. However, one study provided data of functional healing, inflammatory resorption, and replacement resorption [35]; this so-called functional healing, to some degree, was not completely consistent with periodontal healing in the other three studies. And sample sizes were not available for the control group of another study [38]. Thus, only three studies [33, 36,37] were included in the final meta-analysis with moderate heterogeneity (I^2^=68.8%, *p* = 0.040). The pooled results of periodontal healing using a random-effects model indicated EMD applied to the replanted teeth did not significantly promote periodontal healing compared to no EMD at the longest follow-up (RR = 0.38, CI: [0.10, 1.47], *p* = 0.161; Figure 3).

### Risk of extraction after replantation

**Figure 4.**
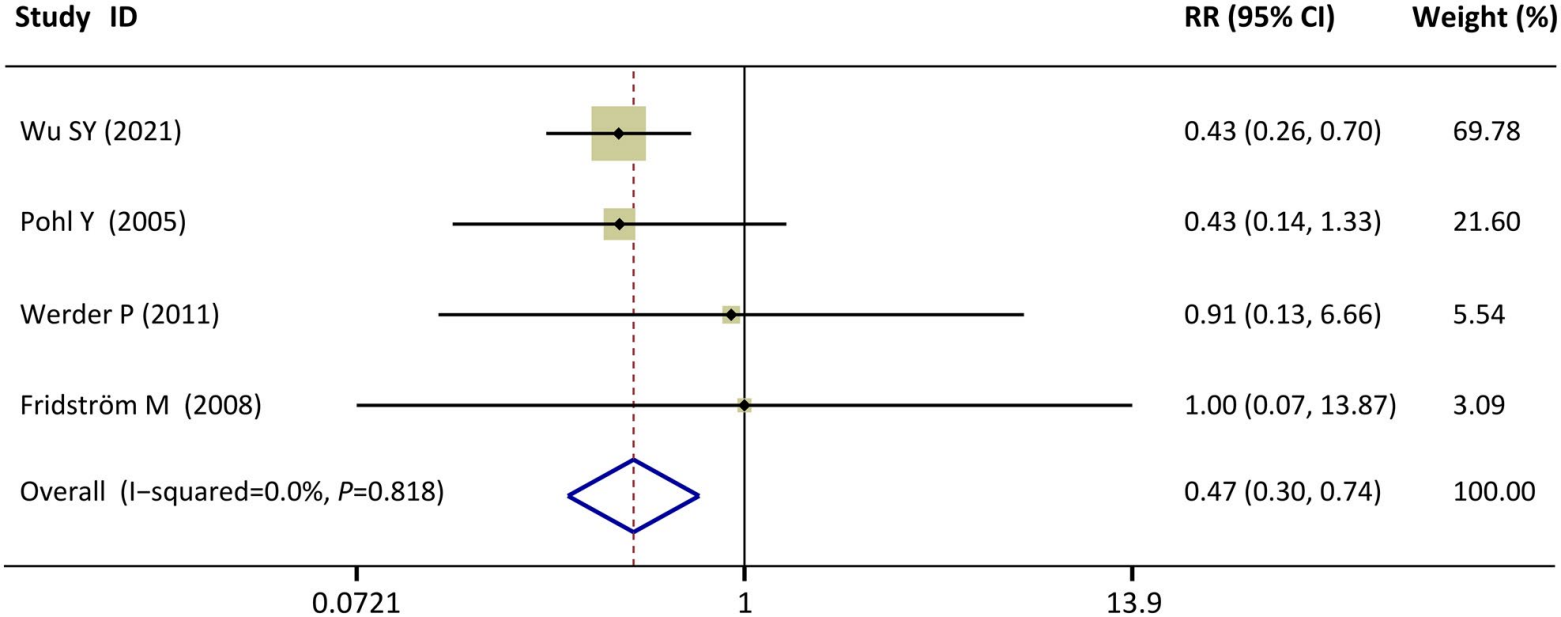
Forest plots of comparisons: EMD versus no-EMD; outcomes: risk of extraction after replantation at the longest follow-up.

The data of extracted teeth or teeth that were diagnosed with “advanced disease” and recommended for extraction after replantation were available in five studies [34–37]. However, one study reported only the total number of teeth extracted without grouping [33]. Therefore, meta-analysis was conducted in four studies [34–37]. The pooled results showed a significantly lower extraction risk in the EMD-treated group than in the non-EMD-treated group (RR = 0.47, CI: [0.30, 0.74], *p* = 0.001; Figure 4). There was no statistical heterogeneity among the included studies (I^2^= 0%, *p* = 0.82).

### Survival rate of the replanted teeth with root resorption during the follow-up period

**Figure 5.**
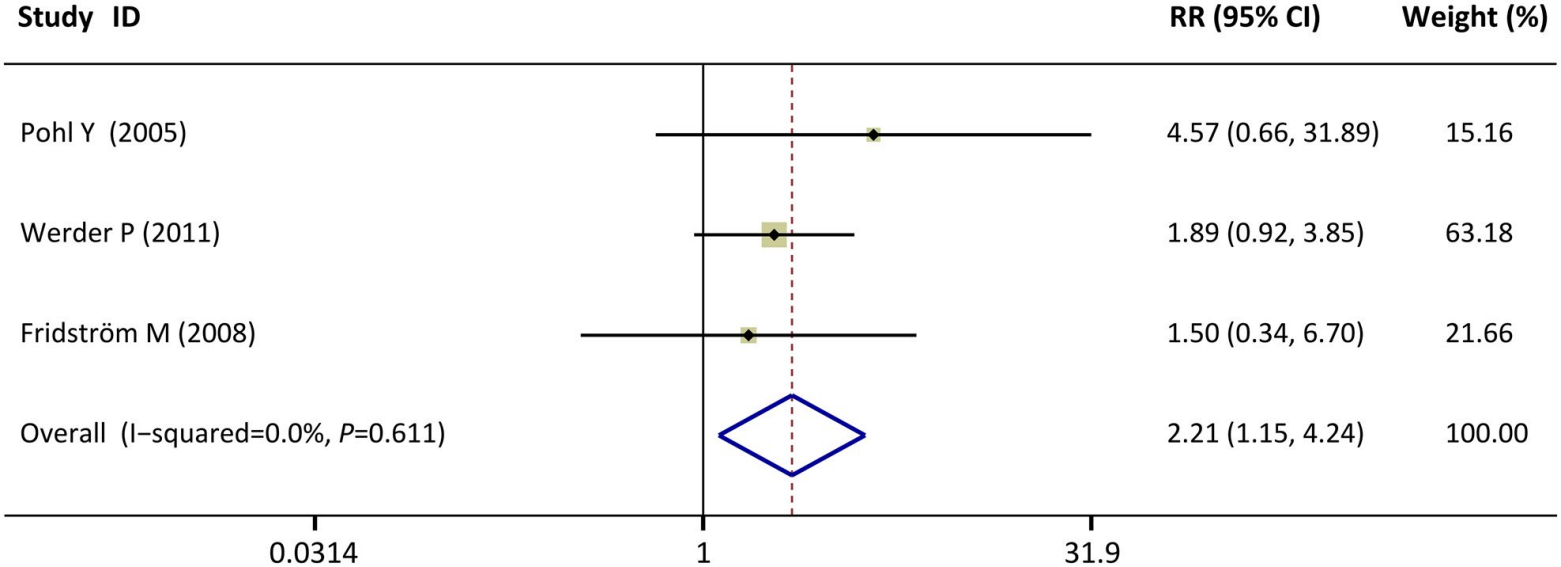
Forest plots of comparisons: EMD versus no-EMD; outcomes: survival rate of the replanted teeth with root resorption during the follow-up period.

The number of replanted teeth with root resorption that survived in the oral cavity during the follow-up was extracted from three studies [35–37]. No statistical heterogeneity was detected (I^2^= 0%, *p* = 0.61). The pooled outcome suggested that the survival rate of replanted teeth with root resorption was significantly greater with the application of EMD than without EMD (RR = 2.21, CI: [1.15, 4.24], *p* = 0.017; Figure 5).

### Types of root resorption after replanting

**Figure 6.**
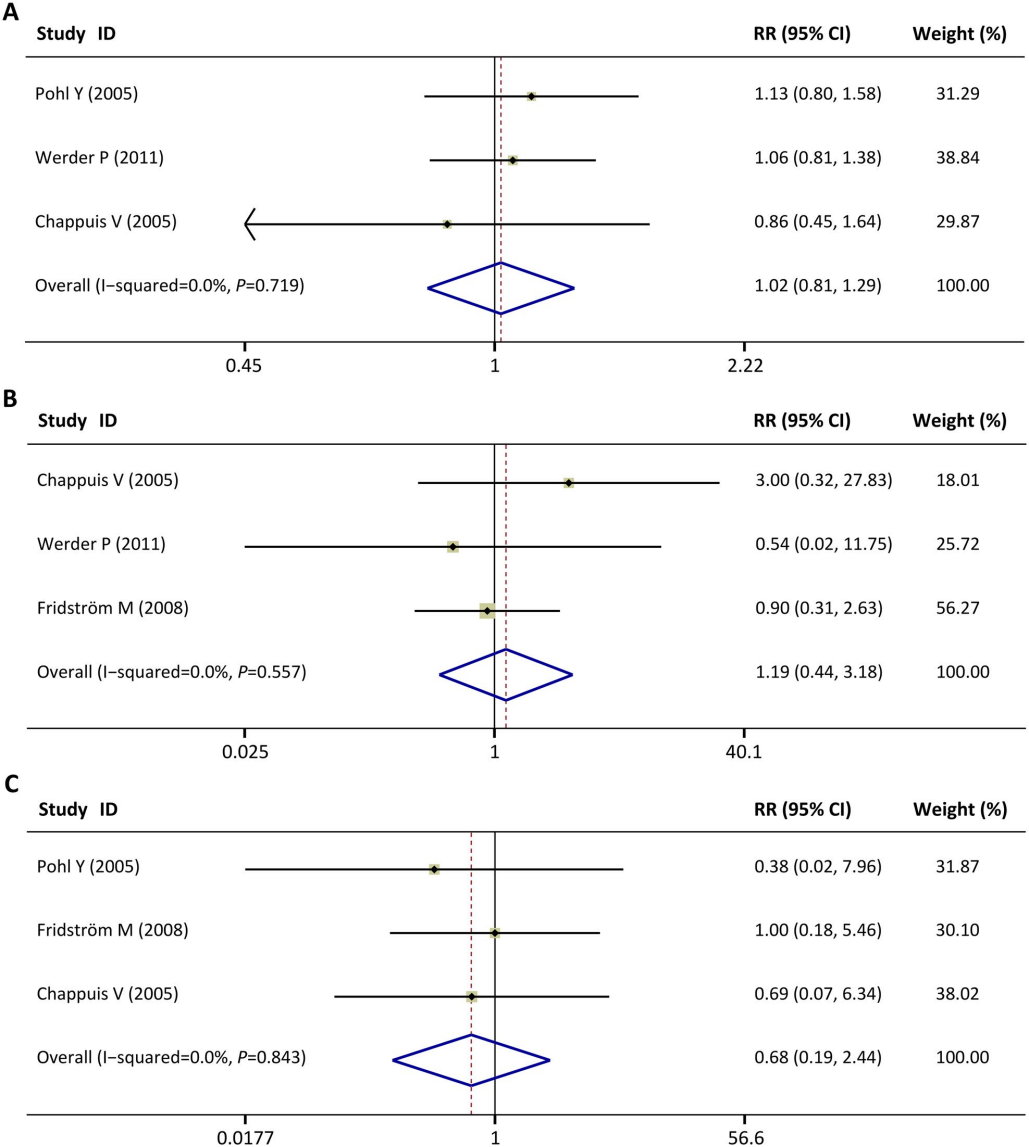
Forest plots of comparisons: EMD versus no-EMD; outcomes: types of root resorption after plantation in resorption cases. (**A**) replacement resorption; (**B**) surface resorption; (**C**) inflammatory resorption.

The root resorption data were available in four studies [33, 35–37]. The types of root resorption were classified as replacement, surface or inflammatory based on the traditional classification. However, not all three types of absorption occurred in all the studies. The pooled outcomes showed no significant difference in the incidence of replacement resorption among the patients with root resorption in the EMD-treated group compared with the non-EMD-treated group (RR = 1.02, CI: [0.81, 1.29], *p* = 0.87; Figure 6A). Heterogeneity among the studies [33, 35,36] did not exist (I^2^= 0%, *p* = 0.72). Our pooled data also demonstrated that EMD treatment tended to increase the incidence of surface resorption in replanted teeth with root resorption (RR = 1.19, CI: [0.44, 3.18], *p* = 0.73; Figure 6B), but this increase was not statistically significant and without heterogeneity (I^2^=0%, *p* = 0.56). In addition, compared with those of the non-EMD group, the pooled data revealed a decrease in the incidence of inflammatory resorption in the EMD-treated group, but this difference did not reach statistical significance (RR = 0.68, CI: [0.19, 2.44], *p* = 0.56; Figure 6C).

### Sensitivity analysis and publication bias

Due to the limited number of included studies, publication bias and sensitivity cannot be effectively evaluated by statistical assessment. Thus, there may be a certain publication bias within the study. To interpret the results more cautiously, we downgraded the level of evidence when considering publication bias.

### Quality of evidence assessment (GRADE)

Since all the enrolled research were nonrandomized clinical trials, the quality evaluation of the combined data began at the ‘LOW’ quality level. All of the pooling outcome suffered from serious impression and limitation of bias. The quality of each pooling outcome was very low.

## Discussion

Avulsion/extraction is the most severe dental trauma and involves the removal of the periodontal ligament, a lack of blood supply to the pulp, and even cementum damage. In general, such events were more prone to appear on maxillary central incisors in growing teenagers with trauma or in patients with periodontally hopeless teeth. Replantation is a treatment option that helps maintain the original dental function in the oral cavity, which is very important for aesthetics and the development of the jawbone [37,38]. The goal of replantation is to achieve physiological healing and reconstruct the original periodontal membrane structure. However, the healing and restoration of replanted teeth are complex. Multiple processes involving multiple pathways that are influenced by various pathophyses. The presence of somatic elements and clinical factors, and not surprisingly, the prognosis of replanted teeth is diverse [39]. Indeed, root absorption, whether surface, inflammatory, or alternative, is frequently observed in the clinic due to the combination of inflammation and trauma. In severe cases, it could result in the inability to retain the affected teeth [2, 40]. The appropriate management of replanted teeth remains a major challenge in routine clinical practice.

Our pooled results showed that EMD applied to patients with replanted teeth did not significantly increase the number of periodontal healing teeth compared to no EMD (RR = 0.38, CI: [0.10, 1.47], *p* = 0.161) at the longest follow-up. According to the split-mouth study of Fridström M et al. EMD treatment did not provide a numerical advantage for producing a normal PDL for replanted teeth. It is, however, important to note that there was a marked qualitative dominance of EMD used, as most of the cases (6 of 9) had a greater degree of root coverage with the attached PDL than with the control teeth when comparing the results within each patient. This is possibly due to the excellent biological effect of EMP, which can significantly promote the adhesion and migration of periodontal ligament cells. Moreover, another important discovery that needs to be highlighted was that a previous study revealed that enamel-related proteins mediate the formation of acellular cementum on the root of the developing tooth, providing a foundation for all necessary tissues associated with functional periodontal attachment [2]. Interestingly, Fridström M et al. verified this phenomenon, as a greater percentage of the relative area covered with cementum was found in EMD-treated teeth. We propose the following speculative explanation. A sufficient number of viable PDL cells is the basis upon which periodontal healing can be achieved. EMD may be unable to reverse the activity of PDL cells with severe damage, thus increasing the amount of periodontal regeneration in replanted teeth. However, when enough living cells are saved, EMD can promote the more extensive coverage of the damaged root surface by the periodontal membrane, thereby achieving better restoration. In animal models, a previous meta–analysis revealed significantly greater PDL healing in EMD-treated groups in the presence of periodontal ligaments; however, only two studies included with high heterogeneity (I^2^=96.895) [21]. Other animal reviews have faced similar situations due to the limited number of studies, species differences and lack of control; thus, no firm conclusions can be drawn [41]. Our present meta-analysis was the first study conducted on clinical trials in which the control and heterogeneity seemed to be low (I^2^=68.8%), but were significant. In some of the included studies [33, 36], EMD was applied in cases with extended dry extraoral storage or a nonphysiologic situation, while cases without EMD used more ideal extraoral storage conditions. Previous research has emphasized that prolonged oral time and the use of a physiologic storage medium have a strong negative effect on the survival of PDL cells in an avulsed tooth [42–44]. During tooth dislocation, PDL cells are isolated from their normal physiological environment and begin to undergo membrane distortion and deformation. The energy stored by the cells begins to be utilized, and after a certain period, the cells will gradually deplete their stored metabolites and lose their viability. Hence, we propose that EMD might still be of value for PDL healing after replantation when given more favourable conditions. The exact effect still needs further exploration with well-designed RCTs for confirmation.

Clinically, the complication of replantation may seriously compromise the longevity of the replanted tooth to such an extent that it may result in its early loss. Encouragingly, our meta-analysis revealed that compared with no EMD, EMD in patients with replanted teeth significantly decreased the risk of extraction at the longest follow-up (RR = 0.47, *p* = 0.001). According to a study by Wu SY, 23.25% of replanted teeth were diagnosed as “diseased” and were recommended for extraction after postoperative assessments. In the EMD treatment group, a lower percentage of “diseased” replanted teeth was observed. This positive result was in line with the study of Pohl Y, where a greater incidence of extraction occurred in patients in whom EMD was not applied to the root surface before replantation. However, in the remaining two studies [36,37], EMD treatment conferred no additional benefit or only slightly decreased the rate of extraction for replanted teeth. Most of the included studies have suggested that root resorption is one of the main contributors to the risk of extraction in patients with replanted teeth [33–37]. That is, strategies to curb the progression of inflammation and resorption will be necessary for replanted teeth with root resorption to survive in the oral cavity.

In our meta-analysis, the survival rate of replanted teeth with root resorption was significantly greater with the application of EMD than without EMD (RR = 2.21, *p* = 0.017). Replacement resorption, a ‘mistake’ because the cells involved in the remodelling of bone cannot distinguish between dental tissues, was the predominant type of posttraumatic external root resorption in most of the included studies [33, 35,36, 38]. The quantity of the remaining replanted teeth involved in replacement resorption was available in two studies [35,36]. Interestingly, the use of EMD has been shown to improve replacement resorption, thus delaying disease progression and reducing tooth extraction in the early stage. During the follow-up period, data from the study of Pohl Y showed that almost all the replanted teeth with replacement resorption (6 in 7) were removed due to the irreversible progression of the disease, and only one survived in the no-EMD group. In contrast, when EMD was used, most of the replacement-resorbed patients (4 in 7) survived in the oral cavity. Similar observations were reported in another study [36]. Replacement resorption occurred in seven teeth with EMD; fortuitously, only one had to be removed, and six teeth were still *in situ*. In contrast, among the replanted teeth without EMD, eleven showed replacement resorption, six were still *in situ*, and five had to be extracted. In addition to replacement resorption, surface and inflammatory resorption should also be considered. Inflammatory resorption, unlike surface resorption that is transient inflammatory absorption with a good prognosis, may progress and result in early tooth loss. Early research generally suggested that infection-related adsorption is derived from the infected pulp space. In avulsion teeth, the displacement of the teeth leads to a disruption of the blood vessels at the apical foramina and to ischaemic pulp necrosis. Additionally, trauma may lead to removal of the root surface and maintenance of microbial stimuli from the infected root canal, providing the necessary continuous stimulation of the resorbing cells. Currently, endodontic treatment can prevent or block inflammatory resorption with a high rate of success. Most of the patients included in the present study ultimately underwent root canal therapy, and inflammatory adsorption occurred in a few patients. In two included studies, there were two cases of infectious absorption in the non-EMD group, and none of them survived. However, in the study by Fridström M, one of the two patients with infection-related teeth treated with EMD survived. The total number of replanted teeth with root resorption survival *in situ* was significantly increased by the use of EMD in the present study (RR = 2.21). Root resorption is due to the dysfunction of periodontal cells, and EMD may have a regulatory effect on this disorder. Cell biology and *in vivo* studies [4–7, 45] have revealed the multiple effects exerted by EMD on PDL cells and tissues, including the inhibition of the inflammatory response and osteoclast maturation in periodontal components; therefore, EMD may preserve the PDL, alveolar bone and cementum. Previous meta-analyses based on animal models have shown that EMD leads to greater surface root resorption and less inflammation and root replacement in the presence of periodontal ligaments. Consistent with this result, clinical research that did not enter the final meta-analysis due to the lack of qualitative information revealed that the EMD protocol was effective at eliminating inflammatory resorption and infection [38]. Here, a question has developed. Did EMD-use determine the type of root absorption on the replanted teeth directly? Our included studies and meta-analyses failed to draw this conclusion. Compared with no EMD applied in root resorption cases, the pooled outcomes showed that EMD increased the incidence of surface resorption and decreased the risk of inflammation resorption; Moreover, the sample sizes were small, and neither of them were statistically significant. Thus, we may prefer to consider that the type of resorption was associated with multiple interacting factors that are mechanically damaged or scraped off, and the treatment of infected pulp has a considerable impact. EMD does not directly change the pattern of  root absorption but may arrest the progression of resorption and improve prognosis because of its anti-inflammatory and bioregulatory effects. Nonetheless, our concept is still in its infancy and is only being explored in a preliminary manner.

The application of EMD has a long history in the field of regenerative medicine. However, the true clinical effects of EMD on the prognosis of replanted teeth are unclear, and this gap in knowledge has impeded the development of therapies. In the present study, we made every attempt to summarize the clinical cases and controls. Although there is insufficient evidence to suggest that EMD can increase the quantity of regenerated periodontal teeth, it can delay absorption and reduce the risk of tooth extraction in the early stage. Namely, replanted teeth might be retained *in situ* for a longer period of time even if there is root absorption. For replanted teeth with no periodontal healing or that achieve functional healing only, there are still some significant implications for preservation. This approach might provide an aesthetically pleasing advantage during the transition period of denture restoration. In addition, the early loss of replanted teeth during adolescence may lead to physiological disruption of normal dentition and jaw development. To date, EMD is still considered a biomaterial with favourable prospects for replantation in clinical applications. Additionally, a better understanding of the application of EMD is still needed in the future, especially when a more ideal situation is present.

There are several limitations to our study. Firstly, there were differences among the time *in vitro*, media for preservation and endodontic treatment of the avulsion teeth in the included studies. The additional use of other medicaments (e.g. glucocorticoids) may also influence the adjunctive effect of EMD by decreasing the initial inflammatory response and resorptive activity due to trauma. Secondly, some studies used EMD in tooth replantation with extended dry extraoral storage or nonphysiologic conditions, while the control was in a more ideal situation, which may underestimate the effect. Thirdly, the sample size of each research was small, which may lack sufficient statistical power to assess the disparities. The pooled results should be interpreted with caution. Fourthly, none of the included studies in this meta-analysis were randomized controlled trial with well design, which might provide a high risk of bias and a low grade of evidence. Thus, future clinical research evaluating the therapeutical effect of EMD on tooth replantation should implement rigorous control or matching measures to eliminate the potential impact of these confounding factors. There is an urgent need for large-scale randomized controlled trials (RCTs).

## Conclusions

The management strategies for patients with replanted teeth have been extremely challenging. Our meta-analysis revealed that compared with no EMD, EMD did not provide a greater advantage in terms of restoring normal PDL healing for replanted teeth. However, it is important to point out that as a biological regulator with multiple functions, EMD may arrest the progression of resorption and improve prognosis. The pooled outcomes suggested that EMD therapy slightly improved the risk of total extraction, and more replanted teeth with root resorption survived in the oral cavity during follow-up.

## Supplementary Material

Supplemental Material

## Data Availability

The data that support the findings of this study are available from the corresponding author, Junbing He, upon reasonable request.
