# Supplementary material for: Enamel matrix derivative in the treatment of tooth replantation: from a biological basis to clinical application
Source: Ann Med. 2024 Nov 9;56(1):2424452. doi: 10.1080/07853890.2024.2424452 (PMC11552275; doi:10.1080/07853890.2024.2424452)
Supplement: Supplemental Material [file IANN_A_2424452_SM8825.zip › Suppl_Data/Additional file 2.docx]

**Additonal file 2. Search Strategy.**

**Potential studies were comprehensively searched via Cochrane Library, Web of Science and PubMed databases from inception to November 23, 2023.**

**1）PubMed (300 records)**

#1 Search: ((Emdogain) OR (enamel matrix protein)) OR (enamel matrix derivative)

#2 Search: ((((avulsion) OR (transplantion)) OR (autotransplantation)) OR (replantation [MeSH Terms])) OR (replanted teeth)

#3 Search: #1 AND #2

**Date run: 2023-11-23**

**Detail of query**

("avulse"[All Fields] OR "avulsed"[All Fields] OR "avulsing"[All Fields] OR "avulsive"[All Fields] OR "fractures, avulsion"[MeSH Terms] OR ("fractures"[All Fields] AND "avulsion"[All Fields]) OR "avulsion fractures"[All Fields] OR "avulsion"[All Fields] OR "avulsions"[All Fields] OR ("transplantability"[All Fields] OR "transplantable"[All Fields] OR "transplantated"[All Fields] OR "transplantating"[All Fields] OR "transplantation"[MeSH Terms] OR "transplantation"[All Fields] OR "transplantations"[All Fields] OR "transplanted"[All Fields] OR "transplanting"[All Fields] OR "transplantation"[MeSH Subheading] OR "transplantation s"[All Fields] OR "transplanter"[All Fields] OR "transplanters"[All Fields] OR "transplantion"[All Fields] OR "transplants"[MeSH Terms] OR "transplants"[All Fields] OR "transplant"[All Fields]) OR ("autotransplantion"[All Fields] OR "transplantation, autologous"[MeSH Terms] OR ("transplantation"[All Fields] AND "autologous"[All Fields]) OR "autologous transplantation"[All Fields] OR "autotransplantation"[All Fields] OR "autotransplantations"[All Fields]) OR "replantation"[MeSH Terms] OR (("replant"[All Fields] OR "replantable"[All Fields] OR "replantation"[MeSH Terms] OR "replantation"[All Fields] OR "replantations"[All Fields] OR "replanted"[All Fields] OR "replanting"[All Fields] OR "replants"[All Fields]) AND ("teeth s"[All Fields] OR "teeths"[All Fields] OR "tooth"[MeSH Terms] OR "tooth"[All Fields] OR "teeth"[All Fields] OR "tooth s"[All Fields] OR "tooths"[All Fields]))) AND ("enamel matrix proteins"[Supplementary Concept] OR "enamel matrix proteins"[All Fields] OR "emdogain"[All Fields] OR (("dental enamel"[MeSH Terms] OR ("dental"[All Fields] AND "enamel"[All Fields]) OR "dental enamel"[All Fields] OR "enamel"[All Fields] OR "enamels"[All Fields] OR "enamel s"[All Fields] OR "enameled"[All Fields] OR "enameling"[All Fields] OR "enamelling"[All Fields]) AND ("altrenogest"[Supplementary Concept] OR "altrenogest"[All Fields] OR "matrix"[All Fields] OR "matrix s"[All Fields] OR "matrixes"[All Fields] OR "matrixs"[All Fields]) AND ("protein s"[All Fields] OR "proteinous"[All Fields] OR "proteins"[MeSH Terms] OR "proteins"[All Fields] OR "protein"[All Fields])) OR (("dental enamel"[MeSH Terms] OR ("dental"[All Fields] AND "enamel"[All Fields]) OR "dental enamel"[All Fields] OR "enamel"[All Fields] OR "enamels"[All Fields] OR "enamel s"[All Fields] OR "enameled"[All Fields] OR "enameling"[All Fields] OR "enamelling"[All Fields]) AND ("altrenogest"[Supplementary Concept] OR "altrenogest"[All Fields] OR "matrix"[All Fields] OR "matrix s"[All Fields] OR "matrixes"[All Fields] OR "matrixs"[All Fields]) AND ("analogs and derivatives"[MeSH Subheading] OR ("analogs"[All Fields] AND "derivatives"[All Fields]) OR "analogs and derivatives"[All Fields] OR "derivatives"[All Fields] OR "derivable"[All Fields] OR "derivant"[All Fields] OR "derivants"[All Fields] OR "derivate"[All Fields] OR "derivated"[All Fields] OR "derivates"[All Fields] OR "derivation"[All Fields] OR "derivations"[All Fields] OR "derivative"[All Fields] OR "derive"[All Fields] OR "derived"[All Fields] OR "derives"[All Fields] OR "deriving"[All Fields])))

**Translations**

**avulsion:** "avulse"[All Fields] OR "avulsed"[All Fields] OR "avulsing"[All Fields] OR "avulsive"[All Fields] OR "fractures, avulsion"[MeSH Terms] OR ("fractures"[All Fields] AND "avulsion"[All Fields]) OR "avulsion fractures"[All Fields] OR "avulsion"[All Fields] OR "avulsions"[All Fields]

**transplantion:** "transplantability"[All Fields] OR "transplantable"[All Fields] OR "transplantated"[All Fields] OR "transplantating"[All Fields] OR "transplantation"[MeSH Terms] OR "transplantation"[All Fields] OR "transplantations"[All Fields] OR "transplanted"[All Fields] OR "transplanting"[All Fields] OR "transplantation"[Subheading] OR "transplantation's"[All Fields] OR "transplanter"[All Fields] OR "transplanters"[All Fields] OR "transplantion"[All Fields] OR "transplants"[MeSH Terms] OR "transplants"[All Fields] OR "transplant"[All Fields]

**autotransplantation:** "autotransplantion"[All Fields] OR "transplantation, autologous"[MeSH Terms] OR ("transplantation"[All Fields] AND "autologous"[All Fields]) OR "autologous transplantation"[All Fields] OR "autotransplantation"[All Fields] OR "autotransplantations"[All Fields]

**replantation [MeSH Terms]:** "replantation"[MeSH Terms]

**replanted:** "replant"[All Fields] OR "replantable"[All Fields] OR "replantation"[MeSH Terms] OR "replantation"[All Fields] OR "replantations"[All Fields] OR "replanted"[All Fields] OR "replanting"[All Fields] OR "replants"[All Fields]

**teeth:** "teeth's"[All Fields] OR "teeths"[All Fields] OR "tooth"[MeSH Terms] OR "tooth"[All Fields] OR "teeth"[All Fields] OR "tooth's"[All Fields] OR "tooths"[All Fields]

**Emdogain:** "enamel matrix proteins"[Supplementary Concept] OR "enamel matrix proteins"[All Fields] OR "emdogain"[All Fields]

**enamel:** "dental enamel"[MeSH Terms] OR ("dental"[All Fields] AND "enamel"[All Fields]) OR "dental enamel"[All Fields] OR "enamel"[All Fields] OR "enamels"[All Fields] OR "enamel's"[All Fields] OR "enameled"[All Fields] OR "enameling"[All Fields] OR "enamelling"[All Fields]

**matrix:** "altrenogest"[Supplementary Concept] OR "altrenogest"[All Fields] OR "matrix"[All Fields] OR "matrix's"[All Fields] OR "matrixes"[All Fields] OR "matrixs"[All Fields]

**protein:** "protein's"[All Fields] OR "proteinous"[All Fields] OR "proteins"[MeSH Terms] OR "proteins"[All Fields] OR "protein"[All Fields]

**enamel:** "dental enamel"[MeSH Terms] OR ("dental"[All Fields] AND "enamel"[All Fields]) OR "dental enamel"[All Fields] OR "enamel"[All Fields] OR "enamels"[All Fields] OR "enamel's"[All Fields] OR "enameled"[All Fields] OR "enameling"[All Fields] OR "enamelling"[All Fields]

**matrix:** "altrenogest"[Supplementary Concept] OR "altrenogest"[All Fields] OR "matrix"[All Fields] OR "matrix's"[All Fields] OR "matrixes"[All Fields] OR "matrixs"[All Fields]

**derivative:** "analogs and derivatives"[Subheading] OR ("analogs"[All Fields] AND "derivatives"[All Fields]) OR "analogs and derivatives"[All Fields] OR "derivatives"[All Fields] OR "derivable"[All Fields] OR "derivant"[All Fields] OR "derivants"[All Fields] OR "derivate"[All Fields] OR "derivated"[All Fields] OR "derivates"[All Fields] OR "derivation"[All Fields] OR "derivations"[All Fields] OR "derivative"[All Fields] OR "derive"[All Fields] OR "derived"[All Fields] OR "derives"[All Fields] OR "deriving"[All Fields]

**2）Web of science (82 records)**

#1 ((TS=(Emdogain)) OR TS=(enamel matrix protein)) OR TS=(enamel matrix derivative) and Preprint Citation Index (Exclude – Database)

#2 ((((TS=(avulsion)) OR TS=(transplantion)) OR TS=(autotransplantation)) OR TS=(replantation)) OR TS=(replanted teeth) and Preprint Citation Index (Exclude – Database)

#3 #1 AND #2

**3）Cochrane Library (2 records)**

**Date run: 2023-11-23**

**Comment:**

**ID Search Hits**

#1 MeSH descriptor: [Replantation] explode all trees 58

#2 avulsion 405

#3 transplantion 63

#4 autotransplantation 574

#5 replanted teeth 21

#6 #1 OR #2 OR #3 OR #4 OR #5 1090

#7 Emdogain 110

#8 enamel matrix protein 82

#9 enamel matrix derivative 292

#10 #7 OR #8 OR #9 360

#11 #6 AND #10 2
