# Supplementary material for: Enamel matrix derivative in the treatment of tooth replantation: from a biological basis to clinical application
Source: Ann Med. 2024 Nov 9;56(1):2424452. doi: 10.1080/07853890.2024.2424452 (PMC11552275; doi:10.1080/07853890.2024.2424452)
Supplement: Supplemental Material [file IANN_A_2424452_SM8825.zip › Suppl_Data/Additional file 3.docx]

**Additional file 3. Quality evaluation of non-RCT**

| **Study ID** | **Selection（4 scores）** | | | |  | **Comparability （2 scores）** | |  | **Outcome（3 scores）** | | | **Total score** |
| --- | --- | --- | --- | --- | --- | --- | --- | --- | --- | --- | --- | --- |
|  | A | B | C | D |  | E | F |  | G | H | I |  |
| Chappuis V, et al. 2005 | * | * | * | * |  |  |  |  | * | * | * | 7 |
| Wu SY, et al. 2021 | * | * | * | * |  |  |  |  | * | * | * | 7 |
| Pohl Y, et al. 2005 | * | * | * | * |  |  |  |  | * | * | * | 7 |
| Werder P, et al. 2011 | * | * | * | * |  |  |  |  | * | * | * | 7 |
| Fridström M, et al. 2008 | * | * | * | * |  | * | * |  | * |  |  | 7 |
| Barrett EJ, et al. 2005 | * | * | * |  |  |  |  |  | * | * | * | 6 |
|  |  |  |  |  |  |  |  |  |  |  |  |  |

Note: "*" is score; In selection, A represents the representative of the exposed queue, B represents the selection of the non-exposed queue, C represents the determination of exposure, and D represents that outcome of interest was not present at start of study; In comparison, E indicated that the study controlled for significant confounders, and F indicated that study controls for any additional factor; In outcomes, G indicated the assessment of outcome, H indicated adequate follow up period for outcome of interest, and I indicated the completeness of follow-up.
